# How does scapula motion change after reverse total shoulder arthroplasty? - a preliminary report

**DOI:** 10.1186/1471-2474-13-210

**Published:** 2012-10-29

**Authors:** Myung-Sun Kim, Keun-Young Lim, Dong-Hyun Lee, David Kovacevic, Nam-Young Cho

**Affiliations:** 1 Department of Orthopaedic Surgery, Chonnam National University College of Medicine, 671, Jebong-Ro, Dong-Gu, Gwangju 501-757, South Korea; 2 Department of Orthopaedic Surgery, Gwangju Hospital, 565-1, Duam-Dong, Buk-Gu, Gwangju, 500-100, South Korea; 3 Department of Orthopaedic Surgery, Cleveland Clinic Foundation, 9500 Euclid Ave., A-41, Cleveland, OH, 44195, USA

**Keywords:** Scapular position, Scapulohumeral rhythm, Reverse Total Shoulder Arthroplasty (RTSA)

## Abstract

**Background:**

Arm elevation is composed of glenohumeral and scapulothoracic motion. Many reports have addressed changes of scapular position across a spectrum of shoulder disease. However, no study has examined changes in scapular position after reverse total shoulder arthroplasty (RTSA). The purpose of this study was to evaluate the changes in scapular position after RTSA compared to patients’ contralateral, nonoperated shoulder.

**Methods:**

Seven patients that underwent RTSA for cuff tear arthropathy from July 2007 to October 2008 were enrolled. The distance between the long axis of the thoracic spine and the inferior pole of the scapula (lateralization of the scapula) was measured on shoulder A-P radiographs at 0 degrees (the neutral position) and at 30, 60, 90, and 120 degrees of shoulder abduction. In addition, the angle between the long axis of the thoracic spine and medial border of the scapula was measured and compared with the patients’ contralateral shoulder.

**Results:**

Scapulohumeral rhythm was 2.4:1 on the operated shoulder and 4.1:1 on the nonoperated, contralateral shoulder at 120 degrees of abduction. The distance between the line of the interspinous process of upper thoracic vertebra and the inferior pole of the scapula showed a negative slope at 0 to 30 degrees abduction on the operated side, but beyond 30 degrees of abduction, this distance showed a more sudden increase than in the contralateral shoulder. The angle between the vertical vertebral line and the scapular medial border also showed greater increase beyond 30 degrees abduction on the operated limb.

**Conclusions:**

The pattern of scapular position after RTSA, was found to differ from that of the contralateral shoulder, and showed a more scapular upward rotation.

## Background

**Figure 1:**
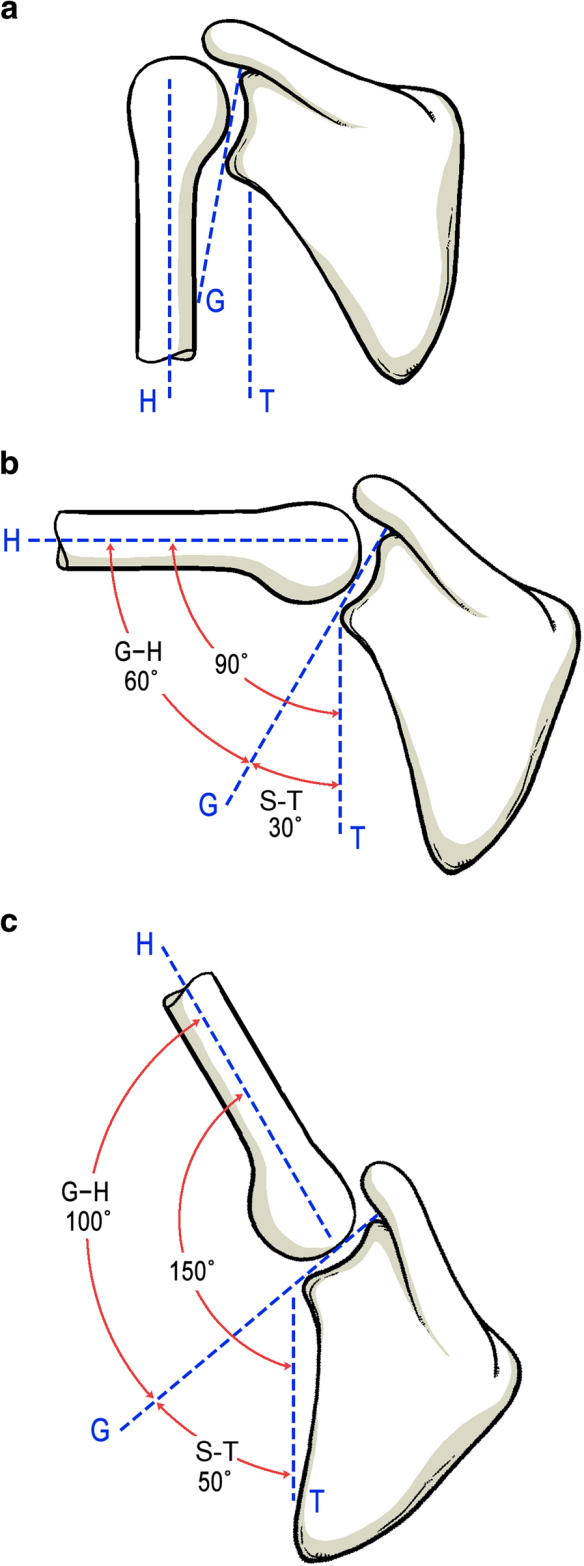
**The schematic drawing of scapulohumeral rhythm.** Resting position (**A**). Position of arm elevation to (**B**) and above (**C**) the horizontal. H = humerus; G = glenoid; T = thorax; G-H = glenohumeral motion; S-T = scapulothoracic motion.

Arm elevation is composed of glenohumeral (GH) and scapulothoracic (ST) joint motion. This was first called scapulohumeral rhythm (SHR) by Codman [1] and the normal ratio was reported by Inman et al. [2] as 2:1 (Figure 1). The motion of the scapula is affected by GH joint motion, and according to Inman, the setting phase is in 0 to 60 degrees of abduction, where the scapula is stabilized. In the setting phase, according to Yano et al. [3], scapular motion is reduced, and beyond 60 degrees, scapulohumeral rhythm is decreased, which means more scapular motion.

Several reports have addressed changes in scapular position in many shoulder diseases, such as, impingement, rotator cuff tear, instability, and frozen shoulder [4-9] while investigation of scapulohumeral rhythm after total shoulder arthroplasty has been performed as well [10]. However, the reverse total shoulder arthroplasty, which is indicated when patients have rotator cuff tear arthropathy or irreparable massive rotator cuff tear with poor motion, biomechanically differs from the native shoulder and anatomic shoulder arthroplasty. Reverse total shoulder arthroplasty medializes the center of rotation, distalizes the humerus, and elongates the deltoid. The lever arm of the deltoid muscle is lengthened so that for any given angular displacement of the humerus, shortening of the deltoid is greater than for total shoulder arthroplasty. Furthermore, no study has previously reported changes in scapular position after reverse total shoulder arthroplasty.

Thus, the purpose of this study was to evaluate the changes in scapular position after reverse total shoulder arthroplasty compared to the nonoperated, contralateral shoulder during abduction in the scapular plane.

## Methods

This study was approved by our institutional review board, and informed consent was obtained from all participants. We reviewed seven primary reverse total shoulder arthroplasties conducted in seven patients between July 2007 and October 2008. The study cohort consisted of two men and five women of mean age 67.4 years (range, 63 to 77 years) at surgery. We included the patient who did not have a history of shoulder disease in the contralateral side, and all had normal active arm elevation and abduction, and active abduction over 120 degrees in operated sides at their follow ups (mean 22.3 months postoperatively). In these seven shoulders, five shoulders had pseudoparalysis with cuff tear arthropathy (cuff tear arthropathy with less than 90 degrees of active forward elevation without actual neurologic deficit) [11], one shoulder had osteoarthritis of the glenohumeral joint and a massive rotator cuff tear, and one shoulder had humeral head osteonecrosis.

All patients were implanted with the Aequalis Reverse Shoulder Prosthesis (Tornier, Edina, MN) with a 6.5 mm stem, a 36 mm glenoid hemisphere, and a 25 mm glenoid baseplate. The component sizes were chosen because they were the most commonly used sizes of the Aequalis Reverse Shoulder system. All patients were placed in the beach chair position and the deltopectoral approach was used to gain access to the shoulder joint. In those cases where the inferior third of the subscapularis tendon was intact, it was released from the lesser tuberosity and preserved for reinsertion at the end of the procedure with transosseous sutures. Using the forearm as a reference, the humeral head osteotomy was performed with a jig to achieve 20 degrees retroversion and 150 degrees inclination. Next, sequentially larger reamers were used to open and prepare the humeral canal. The trial prosthesis was kept in place to protect the proximal humerus during glenoid preparation. The guide wire for the glenoid reamer was positioned so that the glenoid baseplate could be placed as low as possible such that it was flush with the inferior glenoid rim. Reaming was performed manually and cranial and caudal divergently-directed locking screws as well as anterior and posterior non-locking screws were used to secure the glenoid baseplate to provide primary stability. An appropriately sized glenoid hemisphere was then mounted on the baseplate. Polyethylene insert thickness for the humeral component was chosen based on soft-tissue tension during trial reduction. After surgery, patients were placed in an abduction sling for 6 weeks. The initiation of passive range of motion with low intensity supervised physical therapy was started 4 weeks after surgery, and progressed from active assisted range of motion to full active range of motion. Passive external rotation was initially avoided to protect the healing subscapularis tendon repair [12].

**Figure 2:**
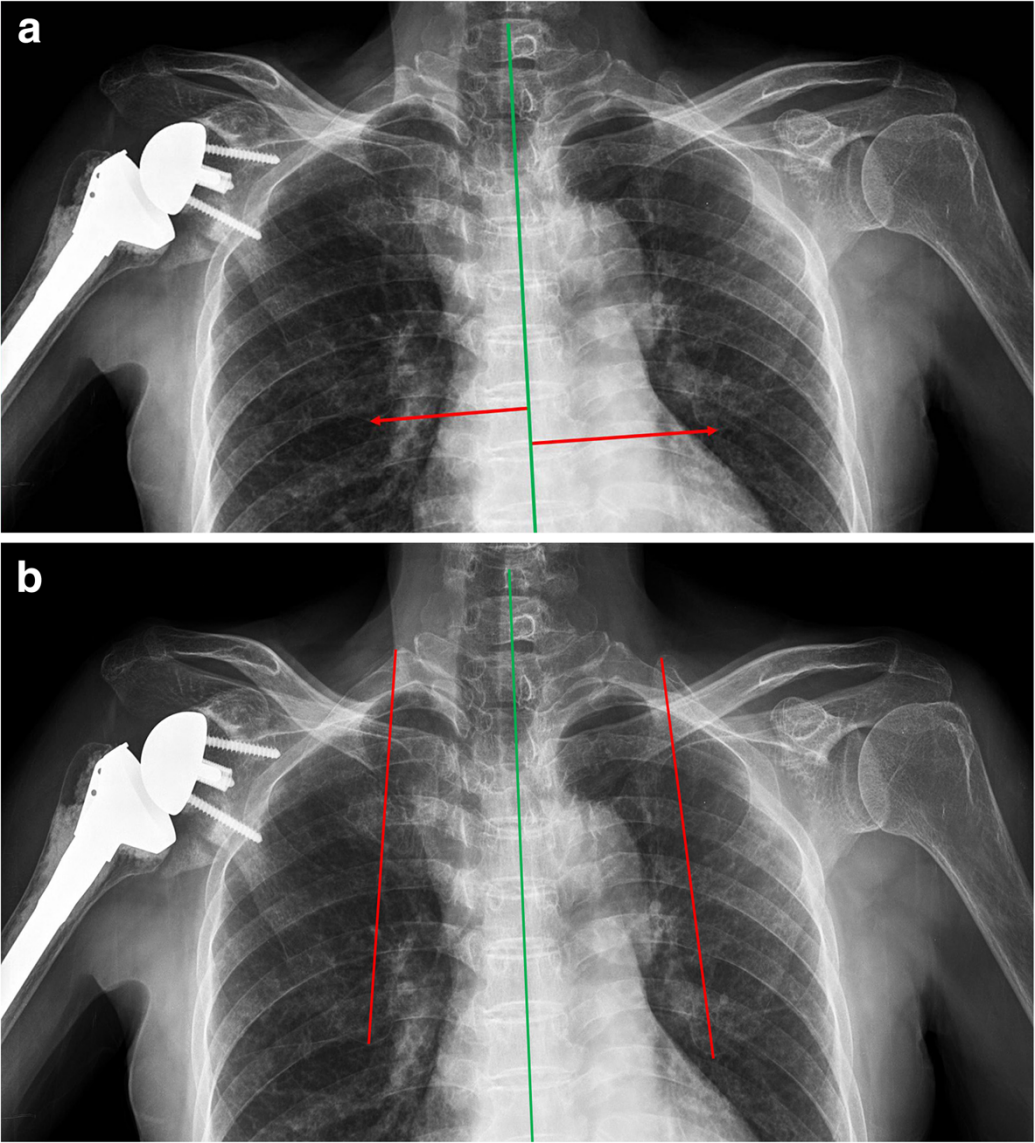
**The measurement method of the distance from the interspinous process of the thoracic vertebra to the inferior angle of the scapula was measured and reported in millimeters (A).** Scapular tilt was measured as the angle formed between a vertical line bisecting the vertebral body and an oblique line drawn from the superior to inferior angle (**B**).

We used two clinical outcome reporting tools to measure pain and function after surgery compared to patients’ preoperative shoulder score. The American Shoulder and Elbow Surgeons (ASES) Shoulder score evaluates pain and activities of daily living reported by the patient. The Korean Shoulder Score (KSS) evaluates function, pain, patient satisfaction, active range of motion in two planes (i.e., forward elevation, internal / external rotation), strength, and endurance. In addition, we measured scapular position at last follow-up by obtaining a standing AP view of the shoulder in the scapular plane at 0, 30, 60, 90 and 120 degrees of abduction. To evaluate scapular position, the distance from the interspinous process of the thoracic vertebra to the inferior angle of the scapula was measured and reported in millimeters (Figure 2A). **S**capular tilt was measured as the angle formed between a vertical line bisecting the vertebral body and an oblique line drawn from the superior to inferior angle (i.e., scapular medial border) (Figure 2B). The scapular position and tilt was determined for each operative shoulder and then compared with that of the contralateral shoulder by consensus agreement amongst the authors (MSK, KYL, DHL, and NYC).

## Results

Mean active forward elevation after surgery was 138.6 degrees, and abduction was 131.2 degrees. The mean visual analog scale score improved from 8.4 preoperatively to 0.8 points postoperatively. The mean ASES shoulder score improved from 30.0 to 83.3 points, and KSS score improved from 37.4 to 76.5 points.

**Figure 3:**
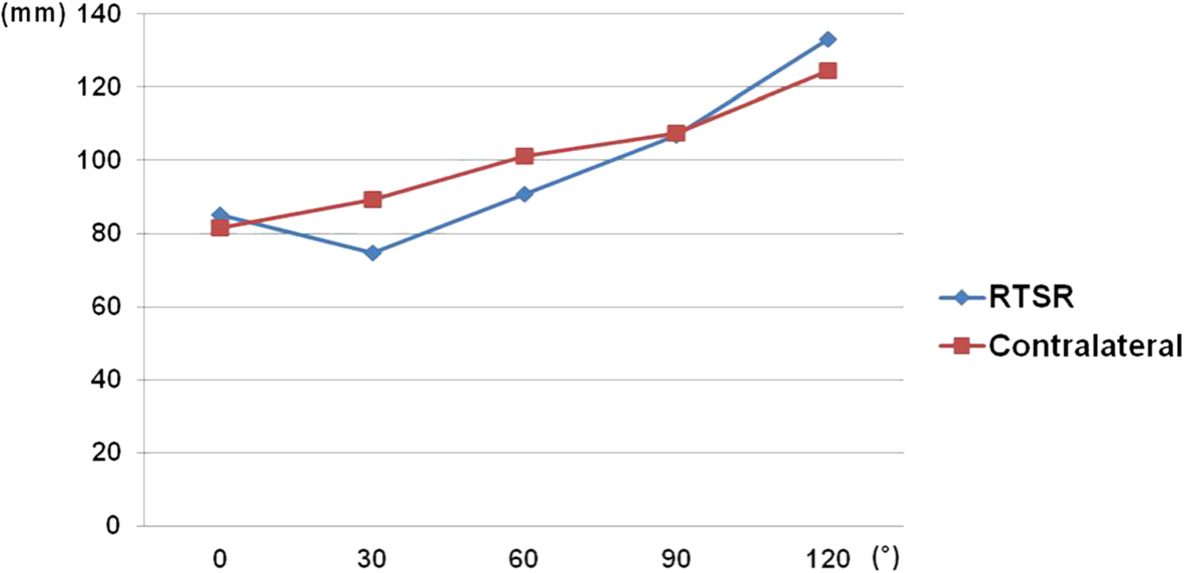
**The distance from the interspinous process of the thoracic vertebra to the inferior angle of the scapula**.

The distances between the interspinous process of the upper thoracic vertebra and the inferior pole of the scapula on operated shoulders were 85.2 mm at 0 degrees, 73.1 mm at 30 degrees, 91.4 mm at 60 degrees, 110.1 mm at 90 degrees and 138.5 mm at 120 degrees of abduction. In the contralateral shoulders, these distances between the vertical vertebral line and the inferior pole of the scapula were 81.4, 90.5, 104.4, 111.8, and 131.1mm at 0, 30, 60, 90 and 120 degrees of abduction, respectively (Figure 3). The difference at each interval of motion was −12.1, 18.3, 18.7, and 28.4 mm for the operated shoulders, and 9.1, 13.9, 7.4, and 19.3 mm for the contralateral shoulders. From 0 to 30 degrees in the abduction arc of motion for the operated shoulders, the slope was negative. However, beyond 30 degrees of abduction, slope of distance showed more sudden increase of distance than the contralateral shoulder (Table 1).

**Table 1:** Differences of the distance* and angle^**†**^**from the line of inter-spinous process of the upper thoracic vertebra at each interval of motion degree of glenohumeral and scapulohumeral rhythm on operated and contralateral shoulder at 30°, 60°, 90°, 120° of abduction**

**Interval**	Distance* (mm)		**Angle**^**†**^**(°)**	
	**RTSA**	**Normal**	**RTSA**	**Normal**
**0°- 30°**	−12.1	9.1	0.8	2.0
**30°- 60°**	18.3	13.9	13.8	6.5
**60°- 90°**	18.7	7.4	6.3	5.8
**90°- 120°**	28.4	19.3	12.6	8.0

RTSA = reverse total shoulder arthroplasty; Distance* = The distance between line of inter-spinous process of the upper thoracic vertebra and inferior pole of scapula; Angle^**†**^ = The angle between line of inter-spinous process of upper thoracic vertebra and scapular medial border.

**Figure 4:**
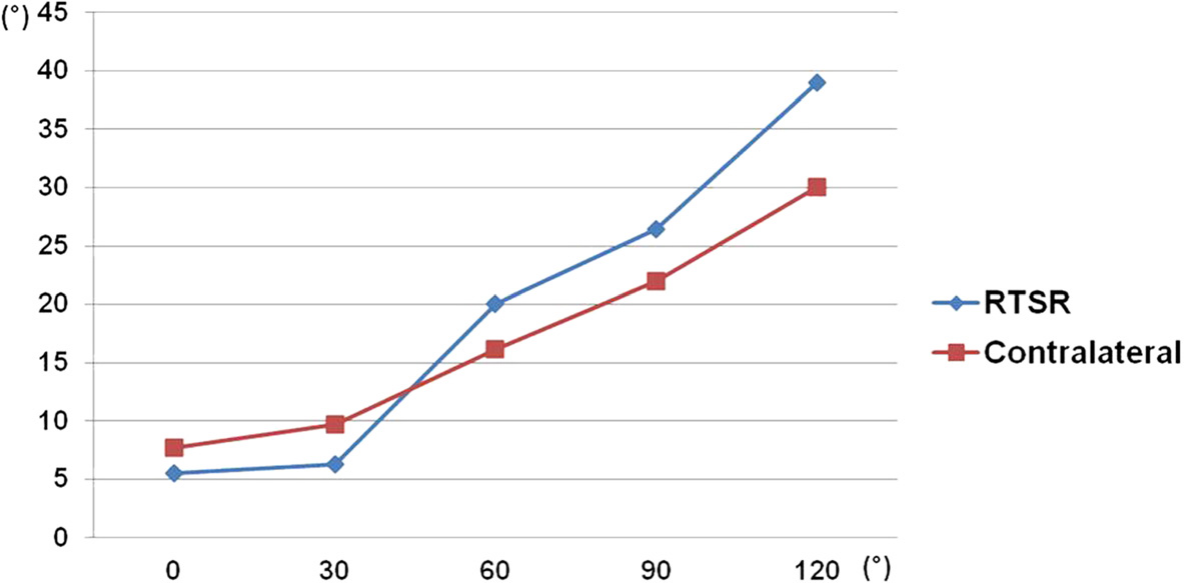
**The angle formed between a vertical line bisecting the vertebral body and an oblique line drawn from the superior to inferior angle**.

The angles between the vertical vertebral line and the scapular medial border on operated shoulders were 5.5, 6.3, 20.1, 26.4, and 39.0 degrees at 0, 30, 60, 90, and 120 degrees of abduction, respectively, and on the contralateral shoulders, these angles were 7.7, 9.7, 16.2, 22.0, and 30.0 degrees, respectively (Figure 4). The difference at each interval of motion was 0.8, 13.8, 6.3, and 12.6 degrees on the operative limb, and 2, 6.5, 5.8, and 8 degrees on the contralateral limb. The slope of the angle also showed more increase beyond 30 degrees abduction after surgery compared to the nonoperative limb (Table 1).

Mean scapulohumeral rhythm was 2.4:1 on operated sides and 4.1:1 on contralateral sides at 120 degrees of abduction, showing less glenohumeral motion and more scapular motion in operated shoulders compared to nonoperated, contralateral shoulders (Table 2).

**Table 2:** The degree of glenohumeral and scapulohumeral rhythm on operated and contralateral shoulder at 30°, 60°, 90°, 120° of abduction

**Abduction**	**RTSA**		**Normal**	
	**G-H**	**S-T**	**G-H**	**S-T**
30**°**	23.8°	0.7°	20.3°	2.0°
60**°**	39.9°	14.6°	43.8°	8.5°
90**°**	63.6°	20.9°	68.0°	14.3°
120**°**	81.0°	33.5°	90.0°	22.3°

RTSA = reverse total shoulder arthroplasty; G-H = glenohumeral motion; S-T = scapulothoracic motion.

## Discussion

Knowledge about scapular movement under the influence of glenohumeral motion aids the understanding of the mechanics and pathology of the shoulder joint. Cathcart [13] was the first to recognize the contribution made by the scapulothoracic joint to normal shoulder complex kinematics. Codman [1] termed this synchronous motion as scapulohumeral rhythm (SHR). Inman et al. [2] reported a SHR of 2:1 in healthy subjects. Since, much research on shoulder kinematics has been directed toward the study of SHR [2,14,15]. Braman et al. [10] reported that shoulder motion differed between patients with advanced glenohumeral osteoarthritis and healthy individuals, and that total shoulder arthroplasty, restored SHRs to normal values.

In the present study, SHR was 2.4:1 on operated shoulders and 4.1:1 on contralateral shoulders at 120 degrees of abduction. This means that there was less glenohumeral motion and more scapular motion in operated shoulders compared to nonoperated, contralateral shoulders. We consider this postoperative change a useful adaptation of scapular mechanics to maintain the tension of the deltoid muscle to generate the forces necessary for shoulder motion, as was mentioned by Mell et al. [7].

In particular, because the function of deltoid muscle is important and the rotator cuff has no function in patients with an implanted reverse total shoulder system, this scapular motion represents a highly meaningful change. However, we carefully propose the hypothesis that that increased scapular motion after RTSR might be able to lead more stretching and fatigue of periscapular muscles at long term follow up after surgery. The specific rehabilitation programs might be needed to be designed to prevent periscapular muscle fatigue after RTSA.

In the present study, during 0 to 30 degrees of abduction, distance between the vertical vertebral line and inferior pole of the scapula was decreased on operated shoulders, which means downward rotation of the scapula on initial abduction; described as motion of the glenohumeral type by Yano et al. [3]. Furthermore, the angle between the vertical vertebral line and scapular medial border increased less than in contralateral shoulders on initial abduction. However, beyond 30 degrees, changes in distance and angle were higher than in contralateral shoulders. Therefore, more scapular upward rotation occurred during middle and late abduction.

Yano et al. [3] proposed two types of upward rotation during the initial phase of elevation. For the glenohumeral type (much glenohumeral motion), the scapula slightly rotates downward and then progresses upward, and for the scapulothoracic type (much scapular motion), the scapula directly rotates upward. Inman et al. [2] used the term ‘the setting phase’ to describe the early phase of shoulder motion during the first 60 degrees of abduction, indicating preparatory stabilization of the scapula to permit controlled humeral motion. Yano et al. [16] reported that SHR was generally greatest (less scapular motion) during the setting phase and that it then decreased beyond 60 degrees of abduction (more scapular motion). Furthermore, the SHR increased again below 60 degrees of abduction. They mentioned that muscular stabilization of the scapula increases while raising the arms, and thus, that less scapular motion is seen during the setting phase.

The present study has some limitations. The most obvious of which is the small number of cases enrolled. This investigation is a pilot study of our early experience with this prosthesis. Based on our inclusion criteria and the fact that it is difficult to have patients return for follow-up, we were only able to include 7 patients at this time. In addition, we only considered upward rotation of the scapula, and not the other planes. Nevertheless, the study has relevance in the context of evaluating changes of scapular upward rotation after reverse total shoulder arthroplasty. Because it is the main movement of the scapula, upward rotation is frequently addressed during treatment and research [17-19]. This preliminary work is a proof of concept study. Future work will include more patients and may be able to use of CT to measure scapular position.

## Conclusions

The pattern of scapular position after RTSA was found to differ between operated shoulders and nonoperated, contralateral shoulders. In particular, there was more upward rotation after surgery.

## Competing interests

The authors declare that they have no competing interests.

## Authors’contributions

MSK designed this study, reviewed the literature and drafted the manuscript. KBL and KYL gave substantial intellectual ideas in drafting the manuscript. NYC participated in the acquisition of data of cases and gave substantial contributetion to interpretation of the literature review. DK gave substantial contributions to revision of manuscript, intellectual ideas, and editorial direction. All authors read and approved the final manuscript.

## Pre-publication history

The pre-publication history for this paper can be accessed here:

http://www.biomedcentral.com/1471-2474/13/210/prepub
